# Evaluation of Generative Language Models in Personalizing Medical Information: Instrument Validation Study

**DOI:** 10.2196/54371

**Published:** 2024-08-13

**Authors:** Aidin Spina, Saman Andalib, Daniel Flores, Rishi Vermani, Faris F Halaseh, Ariana M Nelson

**Affiliations:** 1 School of Medicine University of California, Irvine Irvine, CA United States; 2 Department of Anesthesiology and Perioperative Care University of California, Irvine Irvine, CA United States

**Keywords:** generative language model, GLM, artificial intelligence, AI, low health literacy, LHL, readability, GLMs, language model, language models, health literacy, understandable, understandability, knowledge translation, comprehension, generative, NLP, natural language processing, reading level, reading levels, education, medical text, medical texts, medical information, health information

## Abstract

**Background:**

Although uncertainties exist regarding implementation, artificial intelligence–driven generative language models (GLMs) have enormous potential in medicine. Deployment of GLMs could improve patient comprehension of clinical texts and improve low health literacy.

**Objective:**

The goal of this study is to evaluate the potential of ChatGPT-3.5 and GPT-4 to tailor the complexity of medical information to patient-specific input education level, which is crucial if it is to serve as a tool in addressing low health literacy.

**Methods:**

Input templates related to 2 prevalent chronic diseases—type II diabetes and hypertension—were designed. Each clinical vignette was adjusted for hypothetical patient education levels to evaluate output personalization. To assess the success of a GLM (GPT-3.5 and GPT-4) in tailoring output writing, the readability of pre- and posttransformation outputs were quantified using the Flesch reading ease score (FKRE) and the Flesch-Kincaid grade level (FKGL).

**Results:**

Responses (n=80) were generated using GPT-3.5 and GPT-4 across 2 clinical vignettes. For GPT-3.5, FKRE means were 57.75 (SD 4.75), 51.28 (SD 5.14), 32.28 (SD 4.52), and 28.31 (SD 5.22) for 6th grade, 8th grade, high school, and bachelor’s, respectively; FKGL mean scores were 9.08 (SD 0.90), 10.27 (SD 1.06), 13.4 (SD 0.80), and 13.74 (SD 1.18). GPT-3.5 only aligned with the prespecified education levels at the bachelor’s degree. Conversely, GPT-4’s FKRE mean scores were 74.54 (SD 2.6), 71.25 (SD 4.96), 47.61 (SD 6.13), and 13.71 (SD 5.77), with FKGL mean scores of 6.3 (SD 0.73), 6.7 (SD 1.11), 11.09 (SD 1.26), and 17.03 (SD 1.11) for the same respective education levels. GPT-4 met the target readability for all groups except the 6th-grade FKRE average. Both GLMs produced outputs with statistically significant differences (*P*<.001; 8th grade *P*<.001; high school *P*<.001; bachelors *P*=.003; FKGL: 6th grade *P*=.001; 8th grade *P*<.001; high school *P*<.001; bachelors *P*<.001) between mean FKRE and FKGL across input education levels.

**Conclusions:**

GLMs can change the structure and readability of medical text outputs according to input-specified education. However, GLMs categorize input education designation into 3 broad tiers of output readability: easy (6th and 8th grade), medium (high school), and difficult (bachelor’s degree). This is the first result to suggest that there are broader boundaries in the success of GLMs in output text simplification. Future research must establish how GLMs can reliably personalize medical texts to prespecified education levels to enable a broader impact on health care literacy.

## Introduction

Health literacy is critical for informed health care decisions. However, only 12% of Americans are considered to have proficient health literacy skills [1]. Low health literacy (LHL) is a limited ability to procure, process, and comprehend health information [2]. Importantly, patients with LHL have poorer health outcomes than those with higher health literacy [3]. Many interventions have been proposed and implemented to address health literacy disparities including community health fairs, increased number of primary care visits, and informational handouts [4]. Although the availability of community health fairs and on-demand primary care consultation is variable, the internet is widely accessible [5]. However, internet-derived health information has limitations. Specifically, accessing web-based information and navigating complex user interfaces results in information overload that can negate potential benefits for patients with LHL [6].

Artificial intelligence (AI)–driven chatbots use natural language processing to better interpret and respond to human-like prompts [7]. Generative language models (GLMs), such as ChatGPT (OpenAI), are now regularly used by consumers [8]. Despite the recent increase in the availability of GLMs, implementing AI as a patient education adjunct is not new [9]. Jayakumar et al [10] previously used AI to assist in patient medical education. The incorporation of AI-driven tools resulted in significantly improved decision quality and satisfaction among patients with knee osteoarthritis compared to patients who only received educational material [10]. Given the success of previous iterations of AI in patient education and decision-making, elucidating the potential role of a GLM in a similar capacity could be transformative as a resource to combat LHL [11]. These new tools for patient education can unlock methods for addressing health care concerns, such as pain perception, as illustrated by Sun et al [12], who used education to decrease perceived pain and facilitate recovery.

While there is ostensibly immense potential for this use of AI in health care, at this time, many questions remain, specifically about the accuracy and reproducibility of chatbot-generated medical content [7,13]. While content accuracy is a subject of further clinical discourse, this paper aims to explore the potential of GLMs in tailoring medical text to patient-specific characteristics such as education level. Previous research has evaluated the capability of ChatGPT to simplify a medical text and respond to hypothetical patient questions in various medical specialties [14-17]. In this study, we assessed the ability of ChatGPT versions 3.5 and 4 to transform text to suit a broad range of education levels, including 6th grade, 8th grade, 12th grade, and bachelor’s degree. To elucidate this ability, we tested 2 common clinical scenarios: a patient learning about a diagnosis of diabetes mellitus (DM) or hypertension (HTN). The Flesch reading ease score (FKRE) and the Flesch-Kincaid grade level (FKGL) were implemented as outcome measures as both are clinically validated numeric text assessment tools. The FKRE and FKGL were originally developed to quantify readability ease, with the FKGL being developed specifically for the US Navy in 1975 [18]. As indicated, scores are used commonly, for example, with the US Department of Defense using the FKRE to quantify the readability of its forms and documents [19]. These outcome measures have also frequently been implemented to assess the readability of clinical texts and have been frequently used for assessing outpatient resources [20,21]. Hence, this study explores GLMs as a potentially useful interface to combat LHL by personalizing medical information to a specific education level, as the complexity of clinician-provided and open-access medical information can often inhibit proper understanding. We hypothesized that ChatGPT would be able to successfully create outputs at different readabilities, with GPT-4 being more accurate than its predecessor model, GPT-3.5.

## Methods

### Overview

GPT-3.5 and GPT-4 were used for this study. Both models were developed using reinforcement learning from human feedback, which uses human-generated texts to prompt and train the GLM. This study used a standardized method to generate each input prompt, assess the readability of each output, and perform statistical analyses on the readability scores. This study focused exclusively on evaluating the capacity of GPT-3.5 and GPT-4 to generate outputs with targeted readability levels, without verifying the accuracy of the content produced.

### Input Prompt Creation

A total of 2 input prompts were created that emulate common medical scenarios: DM and HTN. Pertinent information in each input prompt included patient demographics, chief concern at the time of presentation, a set of medical interventions to address the chief concern, and a sentence specifying the desired output (Figures S1-S4 in Multimedia Appendix 1).

Prespecified designation of input patient education level was the focus of this study. Previous research has demonstrated a significant correlation between educational attainment and health literacy, prompting us to use education level as a proxy for health literacy [22,23]. To explore the effect of changing the education level on the generated output, we repeatedly queried the same input prompt while only changing the designated education level of the patient. The education levels included a 6th-grade, 8th-grade, 12th-grade (high school graduate), and a university graduate (bachelor’s degree). Starting at the 6th-grade level ensures alignment with standardized medical recommendations for reading levels of patient-facing materials, while the 8th grade represents the average reading level for an adult in the United States, aligning the study with broad public health guidelines [24-26]. High school graduates were evaluated to bridge the gap between middle and higher education, reflecting a common literacy standard, while the bachelor’s degree level tests the GLM’s ability to tailor complex health information for a more educated audience without unnecessary complexity. Specifically, the high school and bachelor’s levels of education were included as even highly educated people may have LHL due to the complexity of the medical text. Assessing whether GPT-3.5 and GPT-4 can customize outputs for these demographics is essential for validating their use as tools to potentially address LHL.

### Generation of GPT-4 Outputs and Statistical Analysis

In total, 2 sets of input prompts (DM and HTN) were finalized and cataloged in spreadsheets. These input prompts were subsequently entered into GPT-3.5 and GPT-4. Each input scenario—such as GPT-3.5, DM, and 6th grade—was entered into a new conversation window, 5 separate times. All input scenarios were run 5 times to assess reproducibility and to attain statistical significance when comparing groups (Table 1). Next, each output was cataloged, placed into a standardized single-paragraph format, and entered into the readability calculator on Word (Microsoft Corp). The outputs were reformatted into a single paragraph to standardize readability scores, as variations in formatting can affect Word’s ability to accurately measure readability. For this study, the FKRE and the FKGL values were calculated and subjected to statistical analysis [27]. Equations 1 and 2 show how each of these scores are calculated. The FKRE ranges from 0 to 100, with scores of 0 and 100 indicating texts of high and low reading complexity respectively (Table 2).

FKRE = 206.835–1.015 × (total words ÷ total sentences) – 84.6 × (total syllables ÷ total words) **(1)**

FKGL = 0.39 × (total words ÷ total sentences) + 11.8 × (total syllables ÷ total words) – 15.59 **(2)**

Single factor ANOVA was performed to determine if any significant differences existed between the education levels. Once ANOVA confirmed this statistically significant difference, each set of data was subjected to the Tukey multiple comparison post hoc analysis to evaluate differences between the means of each group within each scenario. Significance for all statistical analysis was set at *P*<.05. Single-factor ANOVA with the Tukey post hoc analysis was used because Shapiro-Wilk normality testing and Levene’s test for equality of variances showed that the data did not violate the assumptions of normality or homogeneity of variances. Statistical analyses were performed for individual clinical scenarios and aggregated data. Unpaired 2-tailed *t* tests were also performed to determine the differences in functionality between GPT-3.5 and GPT-4 for both individual clinical scenarios and aggregated data across all 4 education levels. Finally, aggregated analysis was conducted to determine which education level led to outputs with the highest and lowest variation for FKRE and FKGL.

**Table 1:** Summary of scenarios organized by AI^a^ model, grade level, and clinical scenario (N=80).

Clinical scenarios and grade level				Number of scenarios, n (%)	
**GPT-4**					
	**DM^b^**				
		6th grade	5 (6)		
		8th grade	5 (6)		
		High school	5 (6)		
		Bachelor’s	5 (6)		
	**HTN^c^**				
		6th grade	5 (6)		
		8th grade	5 (6)		
		High school	5 (6)		
		Bachelor’s	5 (6)		
**GPT-3.5**					
	**DM**				
		6th grade	5 (6)		
		8th grade	5 (6)		
		High school	5 (6)		
		Bachelor’s	5 (6)		
	**HTN**				
		6th grade	5 (6)		
		8th grade	5 (6)		
		High school	5 (6)		
		Bachelor’s	5 (6)		

^a^AI: artificial intelligence.

^b^DM: diabetes mellitus.

^c^HTN: hypertension.

**Table 2:** Interpretation of the Flesch reading ease score based on the US grade level system.

Score	School level (US)	Description
10.0-0.0	Professional	Extremely difficult to read. Only suitable for university graduates
30.0-10.0	College graduate	Very difficult to read and comprehend
50.0-30.0	College	Difficult to read and comprehend
60.0-50.0	10th to 12th grade	Fairly difficult to read and comprehend
70.0-60.0	8th and 9th grade	“Plain English”
80.0-70.0	7th grade	Fairly easy to read and comprehend
90.0-80.0	6th grade	Easy to read and comprehend. Considered conversational English for speakers
100.0-90.0	5th grade	Extremely easy to read and comprehend

### Ethical Considerations

No application was submitted for review board assessment because no human or animal participants participated directly or indirectly in this study. The University of California, Irvine Institutional Review Board does not require assessment of studies that do not directly or indirectly involve human or animal participants. This study consisted solely of a quantitative evaluation of a GLM for text personalization and is hence exempt from any institutional review.

## Results

### Overview

Descriptive statistics were tabulated for individual clinical vignettes and aggregated data (Tables 3 and 4). Clinical vignette analysis compared how the readability scores changed with education level for each individual clinical case (DM and HTN). When reported for individual clinical vignettes, data have been reported as AI model-clinical case-education level*.* Importantly, 2 readability scores were implemented (FKGL and FKRE), so clinical vignette analysis includes a discussion of how both scores change with education level in each individual clinical example.

In this study, accuracy was defined as a readability score (FKRE or FKGL) whose mean, plus or minus one SD, falls within or below the predefined category. For FKRE, these categories are detailed in Table 2, as originally established by Kincaid et al [18] while for FKGL, the categories are inherently reflected by the corresponding grade levels they represent. For instance, an FKGL score of 6.32 is approximately indicative of a reading level between the 6th and 7th grades. It is important to note that the FKGL formula typically rounds to the nearest whole number, thus for practical purposes, a score of 6.32 is considered appropriate for the 6th grade.

Aggregated data analysis consisted of descriptive statistical reporting similar to the clinical vignette analysis except data were pooled by education level. For example, both of the clinical scenarios were iterated 5 times using “6th-grade” as the prespecified education level. Aggregated data analysis involved pooling the readability scores of all prompt structures that implemented “6th-grade” as the education level (n=10) to observe the consistency of readability scores for educational level across clinical vignettes*.* FKGL and FKRE scores were acquired, so both metrics were implemented in aggregated data analysis.

**Table 3:** Mean and SD of FKRE^a^ and FKGL^b^ for each education level within each clinical vignette.

AI^c^ model	Clinical scenario	Grade level	FKRE, mean (SD)	FKGL, mean (SD)
GPT-4	DM^d^	6th	74.52 (3.12)	6.32 (0.91)
GPT-4	DM	8th	69.42 (3.00)	7.12 (0.91)
GPT-4	DM	HS^e^	47.02 (7.86)	11.4 (1.66)
GPT-4	DM	BS^f^	14.48 (3.33)	16.78 (1.13)
GPT-4	HTN^g^	6th	74.56 (2.34)	6.28 (0.63)
GPT-4	HTN	8th	73.08 (6.17)	6.28 (1.22)
GPT-4	HTN	HS	48.2 (4.69)	10.78 (0.75)
GPT-4	HTN	BS	12.94 (7.89)	17.28 (1.15)
GPT-3.5	DM	6th	54.6 (3.05)	9.7 (0.55)
GPT-3.5	DM	8th	53.6 (6.39)	10.0 (1.19)
GPT-3.5	DM	HS	30.36 (4.81)	13.88 (0.86)
GPT-3.5	DM	BS	26.5 (5.65)	14.44 (1.05)
GPT-3.5	HTN	6th	60.9 (4.08)	8.46 (0.74)
GPT-3.5	HTN	8th	48.96 (2.26)	10.54 (0.98)
GPT-3.5	HTN	HS	34.2 (3.70)	12.92 (0.35)
GPT-3.5	HTN	BS	30.12 (4.63)	13.04 (0.91)

^a^FKRE: Flesch reading ease score.

^b^FKGL: Flesch-Kincaid grade level.

^c^AI: artificial intelligence.

^d^DM: diabetes mellitus.

^e^HS: high school.

^f^BS: bachelor’s degree.

^g^HTN: hypertension.

**Table 4:** Descriptive statistics for FKRE^a^ and FKGL^b^ scores. All 3 clinical scenarios (diabetes and hypertension) scores are aggregated by education level.

AI^c^ model	Grade level	n	FKRE, mean (SD)	FKGL, mean (SD)
GPT-4	6th	10	74.54 (2.6)	6.3 (0.73)
GPT-4	8th	10	71.25 (4.96)	6.7 (1.11)
GPT-4	HS^d^	10	47.61 (6.13)	11.09 (1.26)
GPT-4	BS^e^	10	13.71 (5.77)	17.03 (1.11)
GPT-3.5	6th	10	57.75 (4.75)	9.08 (0.90)
GPT-3.5	8th	10	51.28 (5.14)	10.27 (1.06)
GPT-3.5	HS	10	32.28 (4.52)	13.4 (0.80)
GPT-3.5	BS	10	28.31 (5.22)	13.74 (1.18)

^a^FKRE: Flesch reading ease score.

^b^FKGL: Flesch-Kincaid grade level.

^c^AI: artificial intelligence.

^d^HS: high school.

^e^BS: bachelor’s degree.

### Descriptive Statistics—Clinical Vignette Data

Analysis of each group (ie, AI model-clinical case-education level) revealed that GPT-4 consistently produced accurate average FKRE scores for both DM and HTN scenarios across all education levels, with the exception of the 6th grade, where the FKRE scores were 74.52 (SD 3.12) and 74.56 (SD 2.34), respectively (Table 3). Regarding FKGL measures, GPT-4 achieved the target readability for all education levels except for the bachelor’s degree for the HTN scenario, where the average FKGL was slightly higher at 17.28 (SD 1.15; Table 3). Conversely, GPT-3.5 accurately produced FKRE and FKGL scores that met the required standards only when tasked with generating outputs for bachelor’s degree holders (Table 3). Specifically, in the diabetes scenario at this education level (GPT-3.5-DM-bachelor’s degree), FKRE was 26.5 (SD 5.65) and FKGL was 14.44 (SD 1.05), while in the HTN scenario (GPT-3.5-DM-bachelor’s degree), FKRE and FKGL scores were 30.12 (SD 4.63) and 13.04 (SD 0.91), respectively (Table 3).

The data from clinical vignettes showed that SDs were stable across subgroups for both GPT-3.5 and GPT-4 (Table 3). The average FKRE SD for GPT-3.5 was 4.91 and for GPT-4 was 4.86 (Table 3). The average FKGL SDs were 0.99 for GPT-3.5 and 1.05 for GPT-4 (Table 3). The highest FKRE SD recorded was 7.89 in the GPT-4 HTN-bachelor’s degree scenario, and the lowest was 2.26 in the GPT-3.5 HTN-8th grade scenario (Table 3). For FKGL, the highest SD was 1.66 in the GPT-4 diabetes-high school scenario, and the lowest was 0.35 in the GPT-3.5 HTN-high school scenario (Table 3).

### Descriptive Statistics—Aggregated Data

Data were aggregated for each education level across clinical vignettes as mentioned in the Results Overview section. When aggregated, GPT-4 generated accurate average FKRE scores for most education levels; however, the 6th grade was an exception with an average FKRE of 74.54 (SD 2.60; Table 4). Furthermore, the aggregated data for GPT-4 indicated that the FKGL average was accurate across all tested educational levels (Table 4). In contrast, GPT-3.5 achieved accurate mean FKRE and FKGL scores only at the bachelor’s degree level, with averages of 28.31 (SD 5.22) and 13.74 (SD 1.18), respectively (Table 4).

### ANOVA and the Tukey Post Hoc Analysis

**Figure 1:**
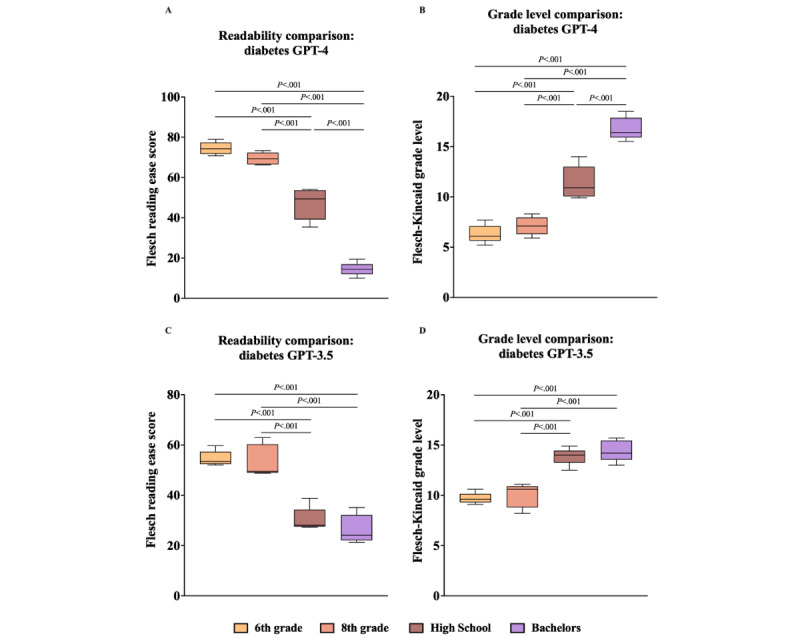
(A) GPT-4 diabetes FKRE, compared with single-factor ANOVA and Tukey post hoc test. (B) GPT-4 diabetes FKGL, compared with single-factor ANOVA and Tukey post hoc Test. (C) GPT-3.5 diabetes FKRE, compared with single-factor ANOVA and Tukey post hoc test. (D) GPT-3.5 diabetes FKGL, compared with single-factor ANOVA and Tukey post hoc test. FKRE: Flesch reading ease score; FKGL: Flesch-Kincaid grade level.

**Figure 2:**
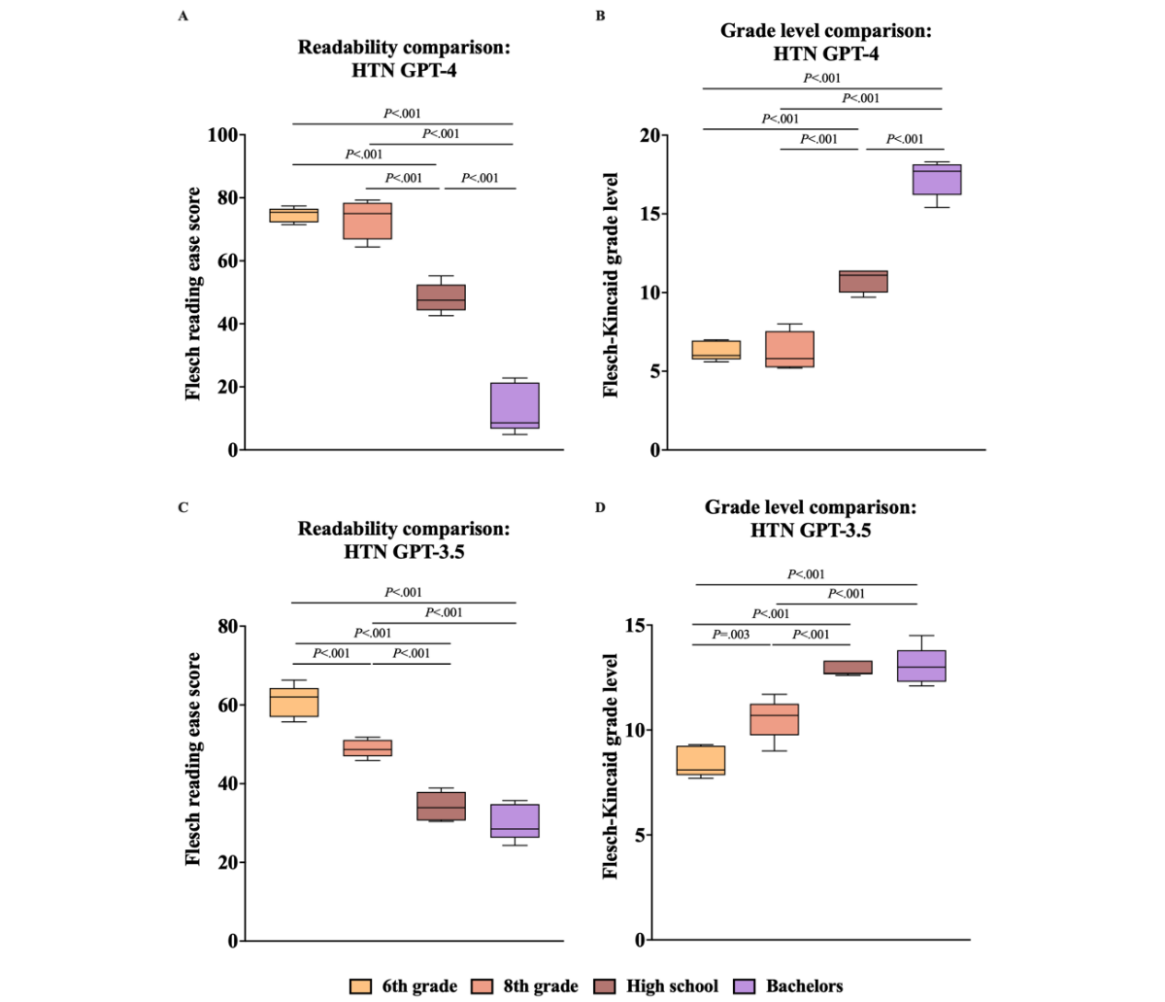
(A) GPT-4 HTN FKRE, compared with single-factor ANOVA and Tukey post hoc test. (B) GPT-4 HTN FKGL, compared with single-factor ANOVA and Tukey post hoc test. (C) GPT-3.5 HTN FKRE, compared with single-factor ANOVA and Tukey post hoc test. (D) GPT-3.5 HTN FKGL, compared with single-factor ANOVA and Tukey post hoc test. FKRE: Flesch reading ease score; FKGL: Flesch-Kincaid grade level; HTN: hypertension.

**Figure 3:**
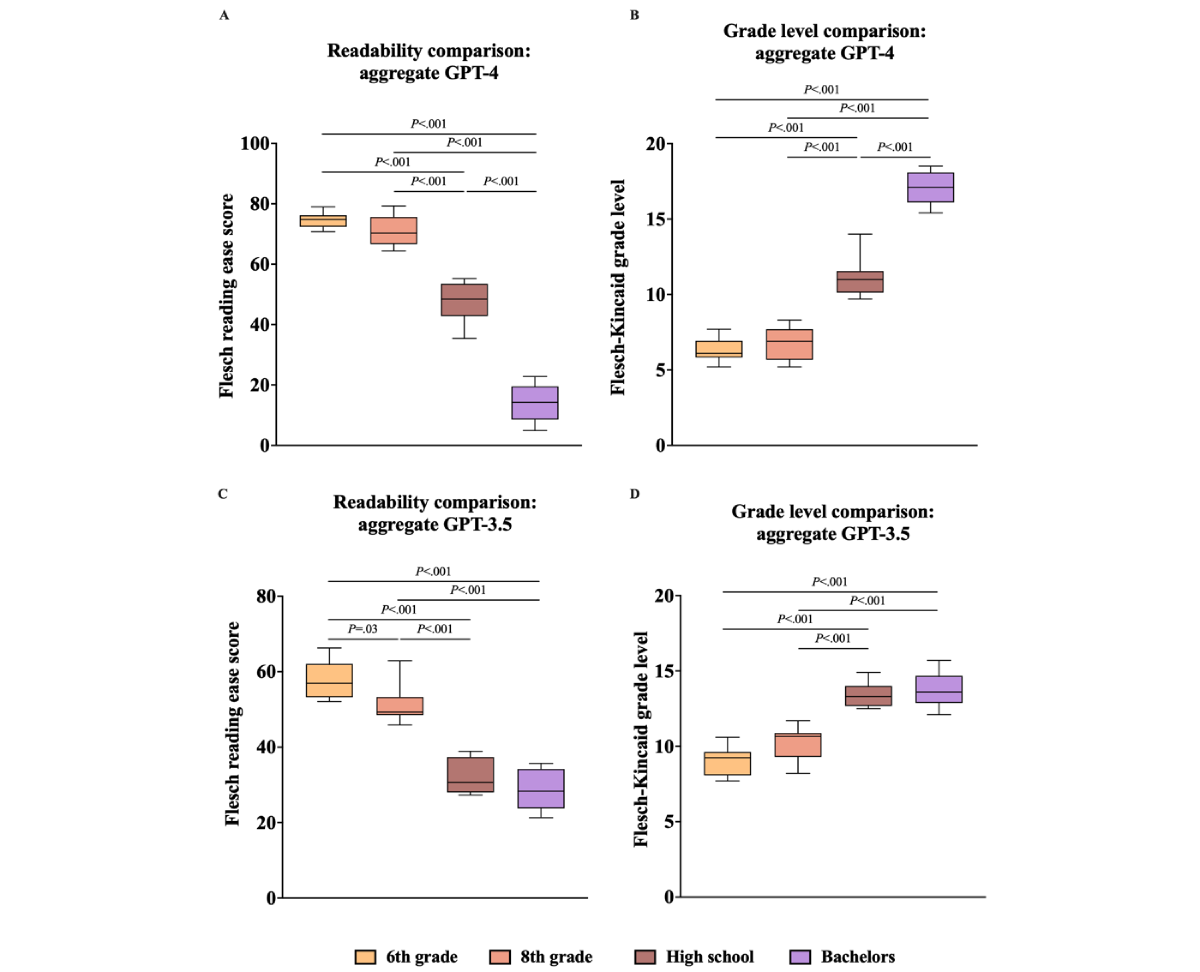
(A) GPT-4 aggregated FKRE, compared with single-factor ANOVA and Tukey post hoc test. (B) GPT-4 aggregated FKGL, compared with single-factor ANOVA and Tukey post hoc test. (C) GPT-3.5 aggregated FKRE, compared with single-factor ANOVA and Tukey post hoc test. (D) GPT-3.5 aggregated FKGL, compared with single-factor ANOVA and Tukey post hoc test. FKRE: Flesch reading ease score; FKGL: Flesch-Kincaid grade level.

To determine the differences between the means of each clinical vignette’s FKRE and FKGL, unidirectional ANOVA and the Tukey multiple comparison post hoc analysis were performed (Figures 1A-3D). The Tukey post hoc analysis showed significant differences between almost all education levels across both clinical vignettes, both individually and when aggregated (Figures 1A-3D). Notably, in the GPT-4 analysis (both individually and aggregated), the only education levels without a statistically significant difference were between 6th grade and 8th grade, for both FKRE and FKGL (Figures 1A, 1B, 2A, 2B, 3A, and 3B). In the GPT-3.5 DM scenario, this pattern persisted, with an additional absence of significance between the high school and bachelor’s education levels for both FKRE and FKGL (Figures 1C, 1D, 2C, 2D, 3C, and 3D). In the GPT-3.5 HTN scenario, the only pair without a significant difference was between high school and bachelor’s degree for both FKRE and FKGL (Figures 2C and 2D). Finally, in the aggregated GPT-3.5 data, FKGL showed no significant differences between 6th-grade and 8th-grade or between high school and bachelor’s degree, while FKRE lacked significance only between high school and bachelor’s degree (Figures 3C and 3D).

### Unpaired 2-Tailed *t* Test Analysis—GPT-3.5 Versus GPT-4

**Figure 4:**
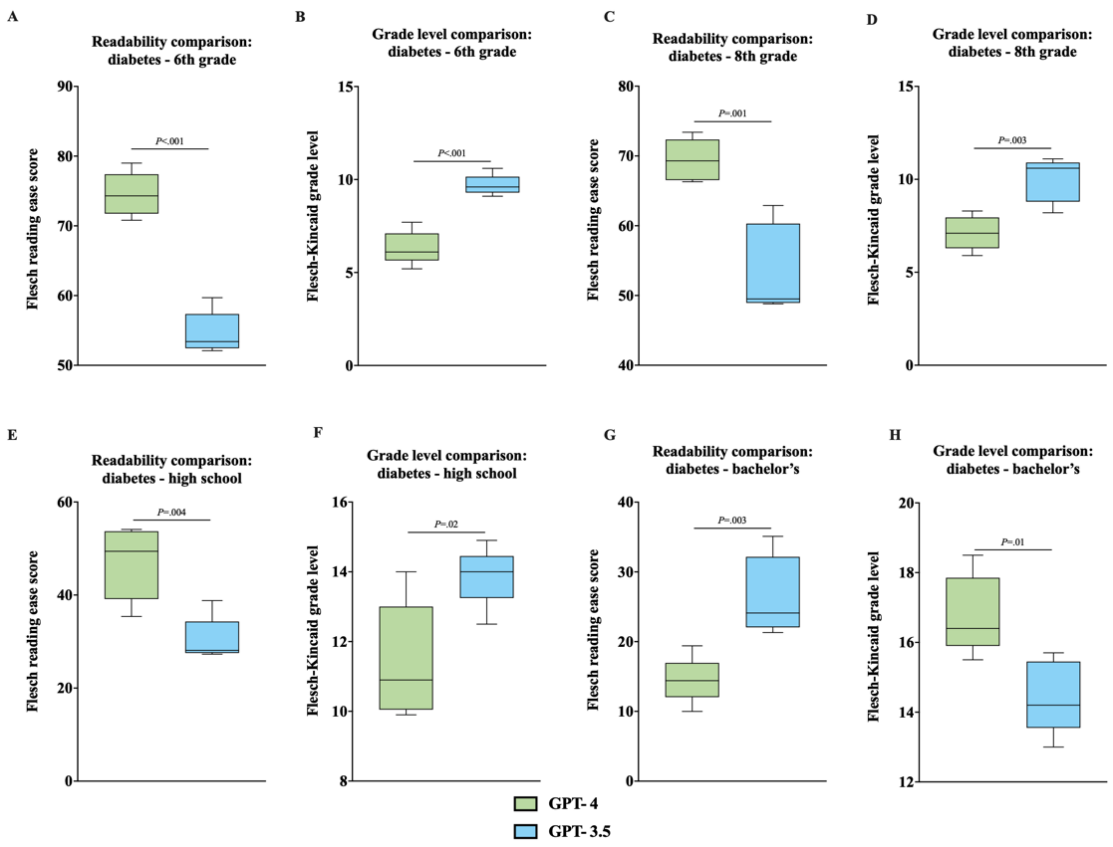
(A) Comparison of FKRE between GPT-4 and GPT-3.5 for diabetes outputs at the 6th-grade level, analyzed with an unpaired 2-tailed *t* test. (B) Comparison of FKGL between GPT-4 and GPT-3.5 for diabetes outputs at the 6th-grade level, analyzed with an unpaired 2-tailed *t* test. (C) Comparison of FKRE between GPT-4 and GPT-3.5 for diabetes outputs at the 8th-grade level, analyzed with an unpaired 2-tailed *t* test. (D) Comparison of FKGL between GPT-4 and GPT-3.5 for diabetes outputs at the 8th-grade level, analyzed with an unpaired 2-tailed *t* test. (E) Comparison of FKRE between GPT-4 and GPT-3.5 for diabetes outputs at the high school level, analyzed with an unpaired 2-tailed *t* test. (F) Comparison of FKGL between GPT-4 and GPT-3.5 for diabetes outputs at the high school level, analyzed with an unpaired 2-tailed *t* test. (G) Comparison of FKRE between GPT-4 and GPT-3.5 for diabetes outputs at the bachelor’s level, analyzed with an unpaired 2-tailed *t* test. (H) Comparison of FKGL between GPT-4 and GPT-3.5 for diabetes outputs at the bachelor’s level, analyzed with an unpaired 2-tailed *t* test. FKRE: Flesch reading ease score; FKGL: Flesch-Kincaid grade level.

**Figure 5:**
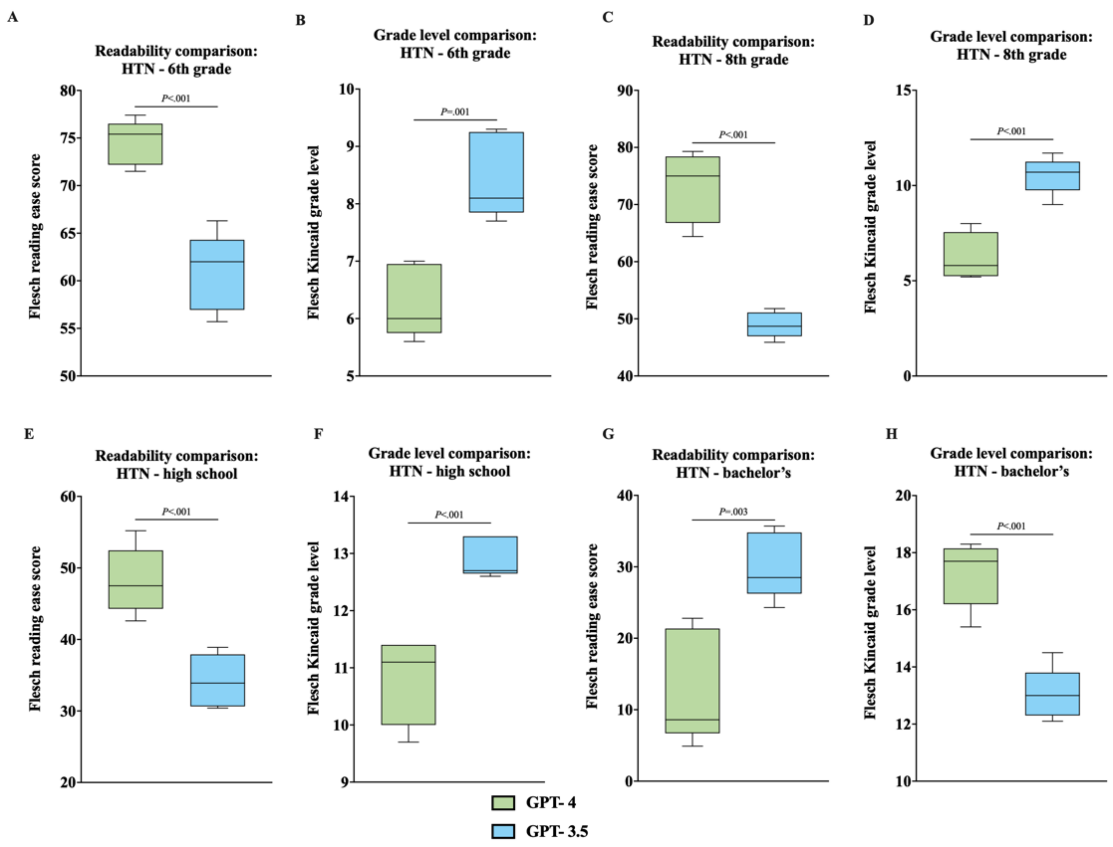
(A) Comparison of FKRE between GPT-4 and GPT-3.5 for HTN outputs at the 6th-grade level, analyzed with an unpaired 2-tailed *t* test. (B) Comparison of FKGL between GPT-4 and GPT-3.5 for HTN outputs at the 6th-grade level, analyzed with an unpaired 2-tailed *t* test. (C) Comparison of FKRE between GPT-4 and GPT-3.5 for HTN outputs at the 8th-grade level, analyzed with an unpaired 2-tailed *t* test. (D) Comparison of FKGL between GPT-4 and GPT-3.5 for HTN outputs at the 8th-grade level, analyzed with an unpaired 2-tailed *t* test. (E) Comparison of FKRE between GPT-4 and GPT-3.5 for HTN outputs at the high school level, analyzed with an unpaired 2-tailed *t* test. (F) Comparison of FKGL between GPT-4 and GPT-3.5 for HTN outputs at the high school level, analyzed with an unpaired 2-tailed *t* test. (G) Comparison of FKRE between GPT-4 and GPT-3.5 for HTN outputs at the bachelor’s level, analyzed with an unpaired 2-tailed *t* test. (H) Comparison of FKGL between GPT-4 and GPT-3.5 for HTN outputs at the bachelor’s level, analyzed with an unpaired 2-tailed *t* test. FKRE: Flesch reading ease score; FKGL: Flesch-Kincaid grade level; HTN: hypertension.

**Figure 6:**
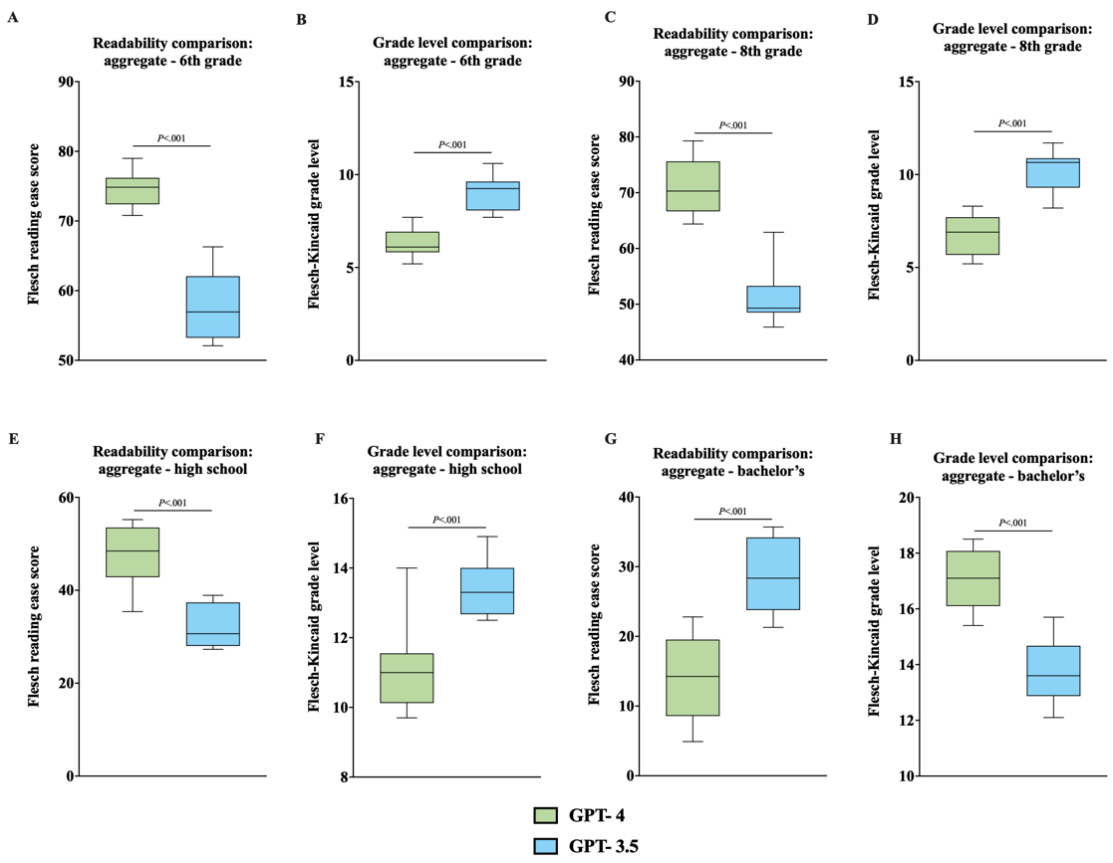
(A) Comparison of FKRE between GPT-4 and GPT-3.5 for aggregated outputs at the 6th-grade level, analyzed with an unpaired 2-tailed *t* test. (B) Comparison of FKGL between GPT-4 and GPT-3.5 for aggregated outputs at the 6th-grade level, analyzed with an unpaired 2-tailed *t* test. (C) Comparison of FKRE between GPT-4 and GPT-3.5 for aggregated outputs at the 8th-grade level, analyzed with an unpaired 2-tailed *t* test. (D) Comparison of FKGL between GPT-4 and GPT-3.5 for aggregated outputs at the 8th-grade level, analyzed with an unpaired 2-tailed *t* test. (E) Comparison of FKRE between GPT-4 and GPT-3.5 for aggregated outputs at the high school level, analyzed with an unpaired 2-tailed *t* test. (F) Comparison of FKGL between GPT-4 and GPT-3.5 for aggregated outputs at the high school level, analyzed with an unpaired 2-tailed *t* test. (G) Comparison of FKRE between GPT-4 and GPT-3.5 for aggregated outputs at the bachelor’s level, analyzed with an unpaired 2-tailed *t* test. (H) Comparison of FKGL between GPT-4 and GPT-3.5 for aggregated outputs at the bachelor’s level, analyzed with an unpaired 2-tailed *t* test. FKRE: Flesch reading ease score; FKGL: Flesch-Kincaid grade level.

When comparing readability scores by education level, unpaired 2-tailed *t* test analysis of individual and aggregated data consistently showed statistically significant differences between GPT-4 and GPT-3.5 (Figures 4A-6H). The analysis revealed that GPT-4 generally produced more readable outputs (higher FKRE and lower FKGL) across all education levels, except for the bachelor’s degree (Figures 4A-6H).

## Discussion

### Overview

Previous investigations into the use of ChatGPT within health care primarily focused on evaluating its potential as an educational tool for patients, particularly in terms of content accuracy and the general readability of its outputs [15,28,29]. Previous studies have also explored the capacity of ChatGPT to distill complex medical information, such as published research abstracts, thereby enhancing accessibility for patients lacking specialized medical knowledge [16,30]. Despite these advancements, no research to date has specifically investigated ChatGPT’s ability to adjust the readability of its outputs to match different educational levels as explicitly directed by users. This study aimed to fill that gap by assessing the capacity of GLMs to produce tailored educational content that adheres to specified readability standards based on user input.

### Principal Results

Analysis of the FKRE data showed some trends that point to GPT-4 having the potential to achieve this goal (Figures 1A-3D). GPT-4 can consistently generate outputs at 3 generalized reading levels: easy (6th and 8th grade), medium (high school), and difficult (bachelor’s degree). In the case of GPT-4, the readability analysis revealed indistinct results exclusively between the outputs for the 6th and 8th grades (Figures 1A, 1B, 2A, 2B, 3A, and 3B). This indistinguishability likely stems from the close progression of these 2 educational stages, being solely separated by the 7th grade. In contrast, all other adjacent educational levels examined in this study were separated by a minimum of 4 grades, which inherently facilitated a more distinct comparison. Although the differences in readability between the outputs for the 6th and 8th grades were not statistically significant, further investigation is warranted to ascertain whether this similarity has any substantive implications for clinical or educational outcomes when these outputs are used in patient education.

Similar to GPT-4, GPT-3.5 also demonstrated nonsignificant differences in readability between the 6th- and 8th-grade levels, both in individual DM scenarios and when considering the overall FKGL. Although the difference in readability scores for the HTN scenario and the combined FKGL scenario was statistically significant between these grades, the scores were higher than the target readability level across the board (Tables 3 and 4 and Figures 2C, 2D, and 3C). One distinct deviation in performance between GPT-3.5 and GPT-4 was the former’s consistent failure to produce outputs with a significant difference in readability between the high school and bachelor’s degree levels across all test cases (Figures 1C, 1D, 2C, 2D, 3C, and 3D). This suggests that GPT-3.5 may be less adept than GPT-4 at differentiating between education levels in its generated text when given specific prompts.

Finally, for the mean FKRE, the trend between education levels was always negative, meaning that as the education level increased, the prompts became harder to read (Figures 1A, 1C, 2A, 2C, 3A, and 3C). Analysis of the FKGL data also showed similarly consistent trends as FKGL average scores always increased with higher education levels (Figures 1B, 1D, 2B, 2D, 3B, and 3D). This is encouraging, as these results show even at this early stage of its existence, GLMs, such as GPT-3.5 and GPT-4, can consistently create outputs of varying readability when explicitly prompted by an input.

GPT-3.5 and GPT-4 demonstrated relatively consistent results in variability across all educational levels, suggesting that both versions of ChatGPT maintain uniform performance irrespective of the complexity of language in the clinical vignettes used. Repeated trials across scenarios—conducted 5 times each—affirmed the reliability of our findings, as consistency in outputs was systematically verified. Notably, the average SDs for the FKRE scores were 4.91 for GPT-3.5 and 4.86 for GPT-4, respectively. Given that these values are less than 5 and considering that a 10-point difference on the FKRE scale roughly corresponds to one grade level (as detailed in Table 1), it can be inferred that 95% of the FKRE scores for both models are expected to cluster within one grade level of each other. This is significant as it highlights the models’ ability to produce outputs with stable readability values, with most variations not deviating dramatically from the mean. Similarly, the average FKGL SDs were 0.99 for GPT-3.5 and 1.05 for GPT-4, indicating that roughly 95% of FKGL scores likely fall within approximately two grade levels, providing further evidence of output consistency. It is important to clarify that this analysis does not assess the accuracy of the outputs in matching the requested readability levels but rather their consistency in reaching said levels.

A key difference in performance between GPT-3.5 and GPT-4 was observed in the accuracy of the outputs’ readability levels. GPT-4 achieved accurate average readability scores in 13 out of 16 scenarios across both FKRE and FKGL, while GPT-3.5 reached accurate average readability scores in only 4 out of 16 scenarios, exclusively at the bachelor’s degree education level (Tables 2 and 3). A comparative grade level analysis using an unpaired 2-tailed *t* test, both for individual and aggregated data, consistently indicated statistically significant differences between GPT-4 and GPT-3.5. This analysis suggests that GPT-4 generally delivered outputs with better readability (higher FKRE and lower FKGL) across various educational levels, with the exception of the bachelor’s degree scenarios (Figures 4A-6H). These findings validate our hypothesis that GPT-4 would outperform its predecessor in output readability accuracy, highlighting its improved language processing capabilities. This suggests that GPT-4 could be more effective in applications requiring nuanced understanding and generation of text such as educational tools or automated content creation. Future research could explore the specific enhancements in GPT-4 that contribute to these improvements and test its performance in other domains to further understand its broader applicability and limitations.

FKRE and FKGL scores were implemented, as they weigh aspects of readability differently (equations 1 and 2). The FKGL emphasizes sentence length more than word length when compared to FKRE [18]. This explains some of the inconsistency in the trend analysis of group variance. Ultimately, our findings concerning FKRE and FKGL scores examine GPT-3.5 and GPT-4’s ability to reliably respond to varying education levels, which as a clinical tool, has the potential to be beneficial in educating patients [31]. However, future research must quantify readability with more metrics to ensure proper personalization of patient-facing educational information.

Our results indicate that GLMs have the potential to create customizable educational materials for patients, suggesting a possible role as a new tool in addressing LHL. Further research is integral in elucidating the capacity that ChatGPT and other GLMs can address LHL, as a patient’s level of health literacy can significantly impact their health outcomes [32]. Specifically, patients with LHL have higher hospitalization rates, are more likely to have poor health status, and have a mortality rate almost double that of patients who do not have LHL [3]. These patients are less likely to receive preventive health services and are more likely to face difficulty accessing the health care they require [33,34]. Current services addressing LHL, including educational pamphlets and community health fairs, have shown limited success due to accessibility constraints [4,35,36]. Thus, attempts to bridge this gap in health literacy and improve health outcomes have been focused on improving health communication techniques for patients with LHL [3]. In this regard, ChatGPT and other new technologies exhibit clear potential, however, are not currently suitable for clinical use in this context. Use of either GPT-3.5 or GPT-4 is not recommended with patients, at the time of this publication due to a few significant limitations.

### Limitations

The major limitation of this study was that it did not analyze the accuracy of the content produced by ChatGPT. Other studies have elucidated the accuracy of ChatGPT outputs in the context of patient queries, particularly within the fields of otolaryngology, urology, and plastic surgery. These studies demonstrated that while ChatGPT can provide accurate answers to patient-style questions, it often answers questions incorrectly [37-39]. Without thorough content validation, the use of GLM technology to generate patient education materials could inadvertently contain false information, potentially leading to the spread of misinformation and resulting in patients mishandling their own care. At present, we strongly recommend that patients seeking medical information obtain their health education materials directly from a qualified physician. Specifically, the use of natural language processors, like ChatGPT, should be restricted to licensed health care providers when disseminating information to patients. This approach ensures the accuracy and reliability of the health information provided. Additionally, this study only tested 2 clinical scenarios. These scenarios centered around a patient using ChatGPT to learn about a new diagnosis of DM or HTN. This study design potentially limits the generalizability of these findings in other clinical contexts.

Another challenge GLMs face is related to maintaining patient privacy [11]. In March of 2023, OpenAI confirmed that they experienced a data leak in which select conversation titles from random users were made visible to other users [11]. The comprehensive impact of this data breach is unclear, but the prospect of future breaches—particularly those involving protected health information—represents a substantial privacy concern for GLMs [11]. At this time, there is no way for a publicly accessible GLM, such as ChatGPT, to be trained on protected health information while maintaining Health Insurance Portability and Accountability Act compliance [40]. Companies and health care professionals looking to develop GLMs for medical use continue to face legal hurdles aimed at protecting patient privacy [41]. In addition to data leaks, ChatGPT was functionally banned for a week in Italy in March of 2023 due to accusations that it was violating European Union data protection laws [42]. This ban prompted other countries, including Germany, Spain, and Canada, to launch investigations into ChatGPT [42]. Given these valid concerns regarding privacy and legality, developers should continue to address these challenges as they integrate this technology into medical care.

### Conclusions

GPT-4 can create outputs within 3 tiers of readability: easy (6th and 8th grade), medium (high school), and difficult (bachelor’s degree). These 3 tiers fall relatively well into their correct intended levels of readability according to the FKRE and FKGL and they allow for preliminary stratification of readability. Unfortunately, GPT-3.5 is less adept at creating customized outputs that fall into their specified readability ranges. Our results highlight GPT-4’s ability to provide patient-centered responses with statistically significant changes to output readability based on education level. Further optimization of this personalization’s accuracy is necessary for it to be an effective clinical tool in addressing LHL. This must be coupled with comprehensive content validation and stringent privacy security measures. The continued evolution of GLMs should provide more robust and capable tools to address these limitations in order to best educate and empower patients.

## Appendix

Multimedia Appendix 1 Revision.

## Abbreviations

AI: artificial intelligence
DM: diabetes mellitus
FKGL: Flesch-Kincaid Grade Level
FKRE: Flesch Reading Ease Score
GLM: generative language model
HTN: hypertension
LHL: low health literacy
