# Supplementary material for: Evaluation of Generative Language Models in Personalizing Medical Information: Instrument Validation Study
Source: JMIR AI. 2024 Aug 13;3:e54371. doi: 10.2196/54371 (PMC11350306; doi:10.2196/54371)
Supplement: Multimedia Appendix 1 [file ai_v3i1e54371_app1.docx]

Figure S1


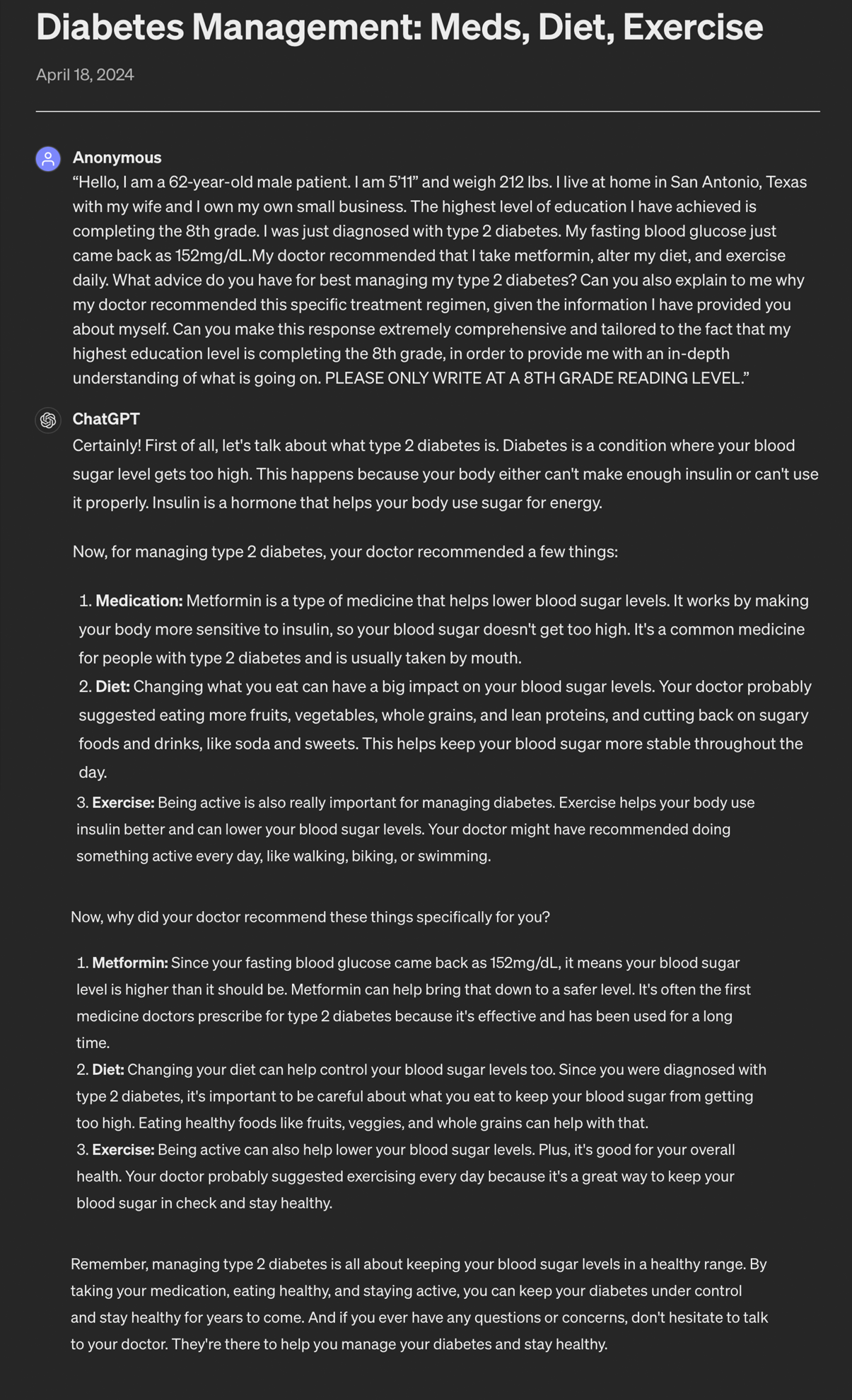


**Figure S1** - Input Prompt and Output for a Patient with Diabetes and an 8th grade education level. Output generated by GPT-3.5 on 4/9/24, <https://chat.openai.com/share/f134d85b-24f3-45c4-a182-f1c5090d5ff7>

FKRE = 62.9

FKGL = 8.2

Figure S2


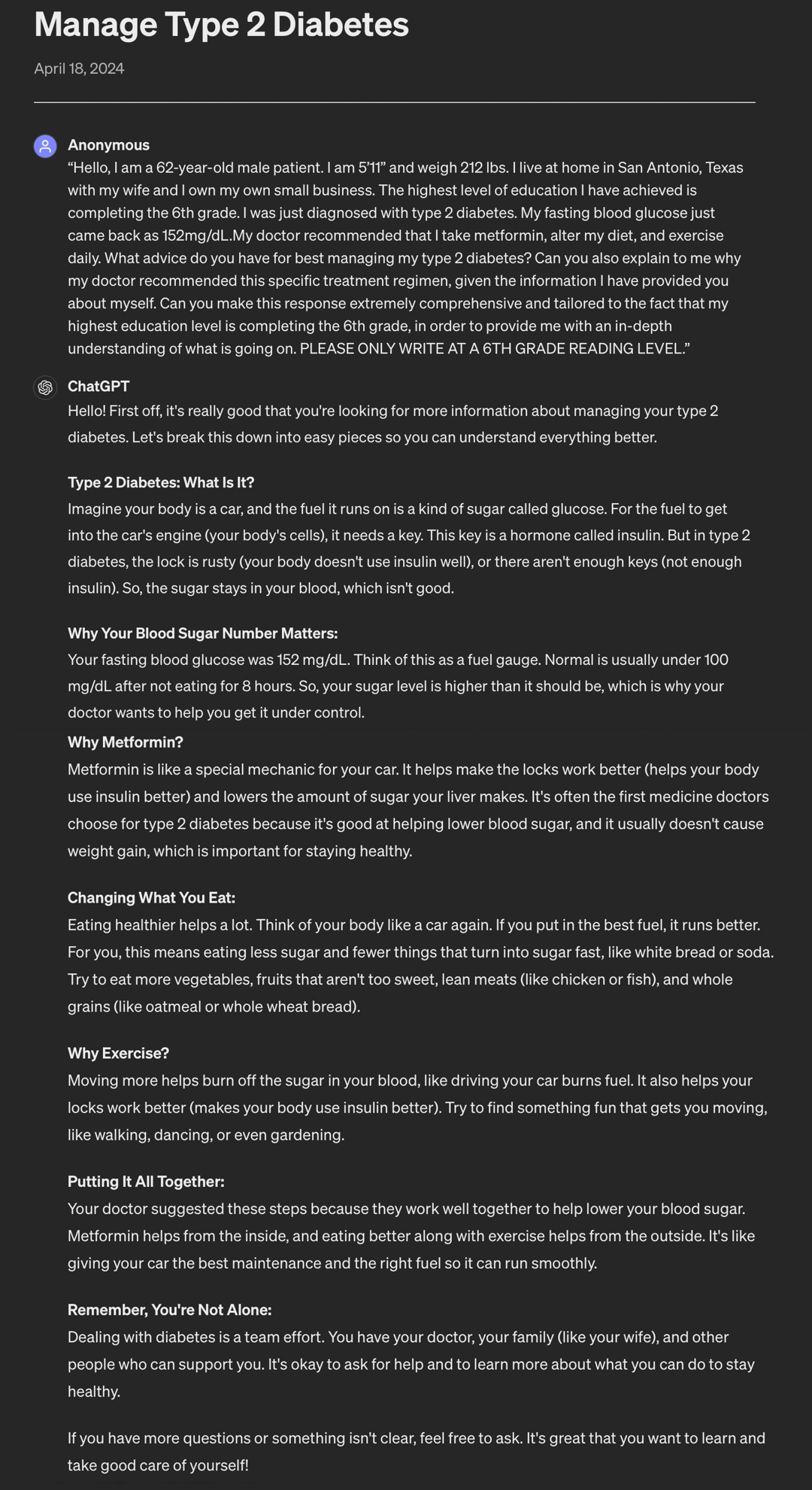


**Figure S2** - Input Prompt and Output for a Patient with Diabetes and an 6th grade education level. Output generated by GPT-4 on 4/9/24, <https://chat.openai.com/share/ccedacec-a0e1-4402-8c43-e60b39ee32ae>

FKRE = 75.8

FKGL = 6.1

Figure S3


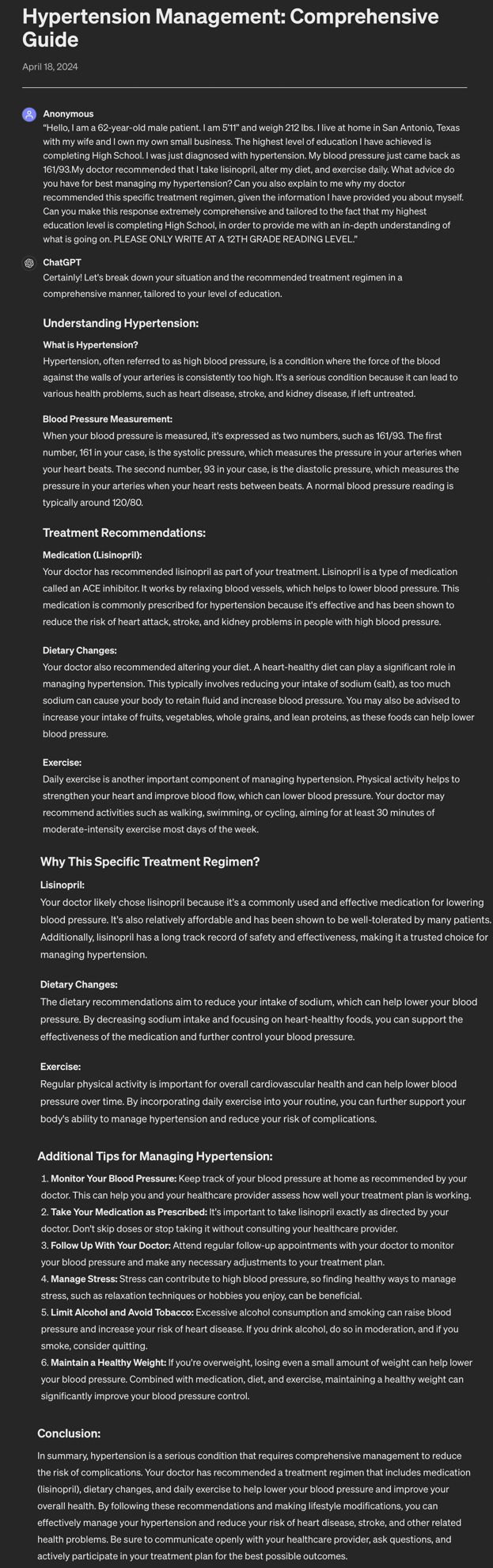


**Figure S3** - Input Prompt and Output for a Patient with Hypertension and a High School education level. Output generated by GPT-3.5 on 4/10/24, <https://chat.openai.com/share/94590af4-38d9-467e-ada8-e717982cff10>

FKRE = 36.9

FKGL = 12.6

Figure S4


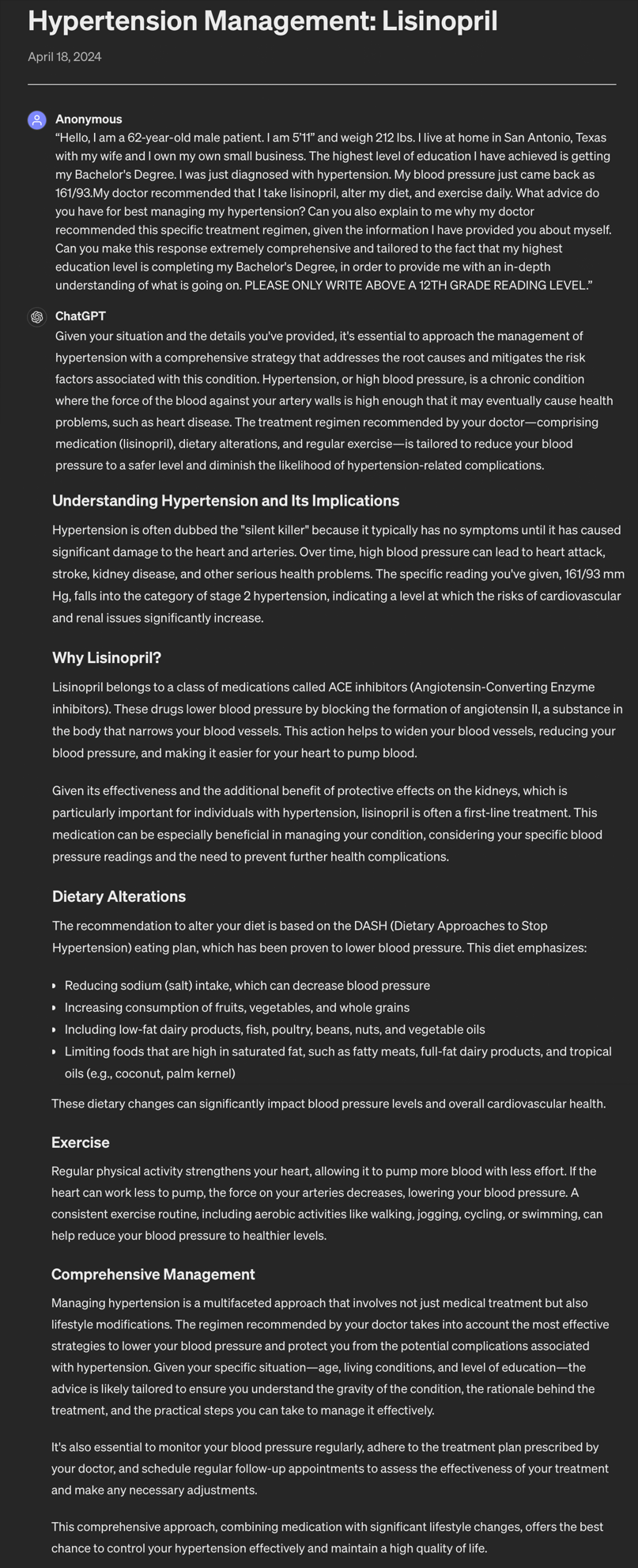


**Figure S4** - Input Prompt and Output for a Patient with Hypertension and a Bachelor’s Degree. Output generated by GPT-3.5 on 4/10/24, <https://chat.openai.com/share/0096da76-b64c-4f3c-bfeb-261cc33146a4>

FKRE = 19.9

FKGL = 17
